# Entire Papilla Preservation Technique with Enamel Matrix Proteins and Allogenic Bone Substitutes for the Treatment of Isolated Intrabony Defects: A 3-Year Follow-Up of a Prospective Case Series

**DOI:** 10.3390/jcm14072374

**Published:** 2025-03-30

**Authors:** Bartłomiej Górski, Sylwia Jakubowska, Beata Wyrębek

**Affiliations:** Department of Periodontology and Oral Mucosa Diseases, Medical University of Warsaw, Binieckiego 6 St., 02-097 Warsaw, Poland; sylwiajakubowska999@interia.pl (S.J.); beatawyrebek@gmail.com (B.W.)

**Keywords:** modified entire papilla preservation technique, intrabony defect, periodontitis, enamel matrix protein, allogenic bone

## Abstract

**Background**: This study aimed to assess the effectiveness of a modified entire papilla preservation technique (MEPPT) for treating isolated intrabony defects in patients with stage III periodontitis. **Material and Methods**: Fifteen patients with 15 interdental intrabony defects were treated with a MEPPT using enamel matrix derivative and allogenic bone. Their probing pocket depth (PPD), clinical attachment level (CAL), gingival recession (GR), keratinized tissue width (KTW), defect depth (DD), full-mouth plaque score (FMPS), full mouth bleeding score (FMBS), radiographic images (radiographic angles, BF and LDF) and intrasurgical parameters were assessed at baseline and 3 years postsurgery. Standardized measurements were taken to evaluate the defect characteristics and treatment outcomes. **Results**: At 3 years, significant improvements from baseline were maintained. Probing pocket depth (PPD) decreased from 7.03 ± 1.61 mm to 3.33 ± 0.89 mm (*p* < 0.0001), clinical attachment level (CAL) improved to 3.08 ± 1.16 mm (*p* < 0.001) and defect depth (DD) decreased from 4.59 ± 1.24 mm to 0.38 ± 0.31 mm (*p* < 0.001). The changes in gingival recession and keratinized tissue were not statistically significant. The results demonstrate sustained clinical stability over a 3-year period. **Conclusions**: Within the limitations of this study, the findings suggest that the modified entire papilla preservation technique (MEPPT) in conjunction with enamel matrix proteins and allogenic bone grafting is an effective approach for the treatment of intrabony defects, leading to statistically significant and sustained clinical improvements over a 3-year period. The study protocol was registered in ClinicalTrials.gov ID NCT05029089.

## 1. Introduction

Currently, the professional mechanical debridement of subgingival plaque using ultrasonic devices and/or hand instruments constitutes a fundamental phase of periodontal therapy. In the majority of cases, non-surgical periodontal treatment effectively maintains periodontal health, reducing the need for surgical intervention [1]. While non-surgical periodontal therapy generally leads to a reduction in pocket depth, in specific clinical scenarios, such as deep vertical bone loss, complete pocket closure may not be attainable [2]. Residual pockets (≥6 mm) associated with intrabony defects constitute a risk factor for periodontitis progression and require surgical therapy [3]. Various regenerative biomaterials and flap designs are employed in periodontal surgery to improve periodontal regeneration [4,5,6,7,8,9,10]. Barrier membranes, either alone or in combination with allogeneic or xenogenic bone substitutes, have been utilized to enhance clot stabilization and provide osteoconductive support for bone regeneration [4]. However, the presence of a membrane may lead to postoperative flap dehiscence, biomaterial exposure and delayed healing [5]. To decrease early wound healing complications, the application of enamel matrix derivative (EMD, Emdogain, Straumann) was implemented to stimulate osteoinduction and regenerate the periodontal ligament and cementum [6].

Several surgical techniques have been developed to enhance periodontal regeneration and to minimize invasiveness in the surgical area. In recent years, the treatment of intrabony defects has increasingly favored minimally invasive surgical techniques aimed at preserving the interdental papilla. In 1985, Takei et al. introduced the papilla preservation technique [7], later modified by Cortellini et al. [8,9,11], with the aim of favoring wound healing, diminishing the risk of flap dehiscence in the interdental area and preserving the soft tissues. The incision at the base of the interdental papilla provides a sufficient view of intrabony defects, although it compromises the vascular integrity of the interdental tissues. Consequently, postsurgical recession, dental hypersensitivity and esthetic problems may occur. In 2017, Aslan et al. [12,13] introduced the entire papilla preservation technique (EPPT). This technique is based on preserving the integrity of the defect-associated interdental papilla through a tunnel-like incision. Therefore, the papilla remains fully supplied by its native, continuous vascular network, effectively preventing wound exposure. Maintaining the papilla intact creates a sealed gingival chamber that stabilizes the blood clot, preserves vascular supply, promotes angiogenesis and enhances the wound healing process. In this technique, vertical incisions are performed, and a full-thickness flap is raised around the tunneled defect-associated papilla. This technique provides significant improvements in clinical outcomes [14]. It was proposed for the treatment of isolated deep and wide intrabony defects that did not involve lingual sites. However, in cases where a higher risk of tearing the interdental papilla occurs, such as for a thin phenotype, narrow interdental space, or fragile interdental papilla with the presence of a crater, the modification of the EPPT offers advantages. This modification involves extending the buccal flap mesiodistally to expose the cortical bone of adjacent teeth, thereby enhancing access to the surgical site, providing less tension on the flap while still preserving the papilla. Additionally, to improve visualization and access to a single-wall intrabony defect, the vertical releasing incision is lengthened as needed. Therefore, the aim of this study was to evaluate the clinical and radiographic outcomes of a modified EPPT (EPPT with the elevation of the larger buccal flap) in combination with EMD and radiation-sterilized, allogeneic bone substitution in the treatment of isolated intrabony defects.

## 2. Materials and Methods

Participants: Patients diagnosed with stage III periodontitis at the Department of Periodontology of the Medical University of Warsaw who fulfilled the inclusion criteria were included. Non-surgical periodontal treatment was conducted and reevaluated after 3 months. The patients were informed of the procedure, potential risks and the benefits of their participation in the study. All signed consent forms. Inclusion criteria: 1. diagnosis of stage III periodontitis [15]; 2. no systemic diseases; 3. no use of medications affecting the periodontal status; 4. non-smokers; 5. neither pregnant nor lactating; 6. presence of at least one tooth with PPD ≥ 6 mm, CAL ≥ 5 mm and bone defect depth (5DD) ≥ 3 mm as detected in periapical radiographs; 7. full-mouth plaque score (FMPS) ≤ 20% and full-mouth bleeding score (FMBS) ≤ 20%; 8. tooth has to be vital or properly treated; 9. no furcation involvement.

Study Design: The clinical measurements were taken by the same experienced examiner. FMPS was calculated as the percentage of tooth surfaces that exhibited plaque [16] and FMBS as the percentage of periodontal pockets that bled from the bottom 15 s after careful probing [17]. The clinical parameters were registered with a millimeter periodontal probe (15 mm University of North Carolina (UNC) probe, Hu-Friedy, Chicago, IL, USA) as follows: 1. PPD as the distance from the gingival margin to the base of the periodontal pocket at six points per tooth (i.e., distobuccal, buccal, mesiobuccal, distolingual, lingual and mesiolingual); 2. CAL as the distance from the cemento-enamel junction (CEJ) to the base of the periodontal pocket at six points per tooth; 3. gingival recession (GR) as the distance from the CEJ to the gingival margin at the mid-buccal point of the tooth; 4. keratinized tissue width (KTW) as the distance from the gingival margin to the mucogingival junction at the mid-buccal point of the tooth after staining with an iodine solution. The clinical measurements were taken immediately before and 3 years after periodontal surgery.

The following intrasurgery measurements were recorded upon the completion of debridement during surgery: (1) defect depth as the distance between the bottom of the defect and the most coronal point of the bony walls surrounding the defect; (2) defect width as the distance from the most coronal point of the bony walls surrounding the defect to the root surface; (3) the number of remaining walls of the defect (defects were classified as one-wall, two-wall and three-wall defects).

Radiographic measurements: Standardized digital periapical radiographs were collected from each patient with film holders and a paralleling technique using an X-ray unit operating at 70 kV, 4 mA and a 0.1 s exposure time prior to surgery and 3 years postoperatively. The radiographs were analyzed using Planmeca Romexis Viewer software (Planmeca, Helsinki, Finland). Anatomic landmarks, which included the CEJ, alveolar crest (AC) and base of the defect (BD), were selected on the radiographs. Two auxiliary lines were drawn, the first in the tooth axis (AUX1) and the second line (AUX2) from the AC, perpendicular to AUX1. Defect depth (DD) was measured as the distance from the spot where AUX2 crossed the CEJ-BD line to the base of the defect. The radiographic defect angle was calculated between the intersection of the CEJ-BD line of the tooth and the delimitation of the wall of the defect [18]. During the follow-up analysis, the radiographic bone-fill (BF) was quantified utilizing the Planmeca Romexis Viewer software program as the percentage of bone-fill, depicting the change in bone level as compared to the preset landmarks and initial radiograph. Linear radiographic measurements were taken of the present landmarks using the Planmeca Romexis Viewer software program, which provides a computerized ruler, and were compared with the initial situation.

Surgical Procedures: All surgical procedures were performed by one surgeon (BG) using surgical loupes (×3.3). The anesthetic of choice was 4% articaine hydrochloride with adrenaline (1:100,000) (Ubistesin Forte 1.7 mL, 3-M ESPE, Saint Paul, MN, USA) based on its reported superiority over local anesthetic agents in oral surgical procedures [19,20]. After local anesthesia, a buccal intra-crevicular incision of the defect-associated tooth and the adjacent tooth was performed. Subsequently, a short buccal vertical incision positioned contralaterally to the intrabony defect was extended beyond the mucogingival line. A buccal full-thickness flap was elevated, extending from the vertical incision to the defect-associated papilla and to the contralateral site of the whole width of the adjacent tooth. The interdental papilla was carefully elevated in a full-thickness manner to the coronal edge of the lingual bone crest. The intrabony defect was meticulously debrided and the roots were carefully planned. The surgical area was rinsed with sterile saline and the exposed root surfaces were conditioned for 2 min with 24% Ethylenediaminetetraacetic acid (EDTA) (PrefGel^®^, Straumann, Basel, Switzerland) and then thoroughly rinsed with sterile saline. Subsequently, enamel matrix derivative (EMD) (Emdogain, Straumann) was applied on the exposed and air-dried root surface. In the next step, EMD mixed with frozen, radiation-sterilized, allogenic bone granules consisting of cortical and cancellous bone prepared by the Department of Transplantology and Central Tissue Bank, Medical University of Warsaw, was placed into the defect [21]. The suturing approach consisted of a single sling suture (Seralon 6/0 12 mm 3/8) at the buccal site of the adjacent tooth and simple sutures in vertical incisions (Seralon 7/0 10 mm 3/8).

Postsurgical Care: The patients received postoperative instructions and were asked to avoid brushing, flossing and chewing in the treated area for 2 weeks. Patients were advised to rinse their mouth with 0.12% chlorhexidine three times a day for 4 weeks. At week 2, the sutures were removed and patients resumed careful brushing with a soft toothbrush. The patients were placed on a 2-week recall system for 3 months and every 2 months for 6 months [22].

**Figure 1:**
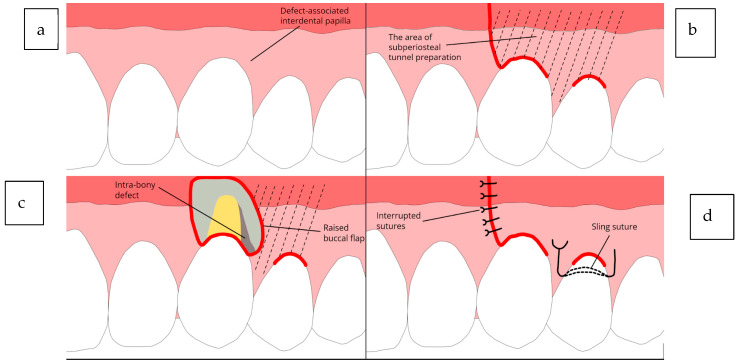
Diagrams illustrating the EPPT. (**a**) Intrabony defect affecting the maxillary left canine. (**b**) Flap design with a single vertical incision on the buccal side along with two sulcular incisions. (**c**) Elevation of the buccal flap. (**d**) Primary closure of the vertical incision using simple interrupted sutures and a sling suture at the neighboring tooth.

**Figure 2:**
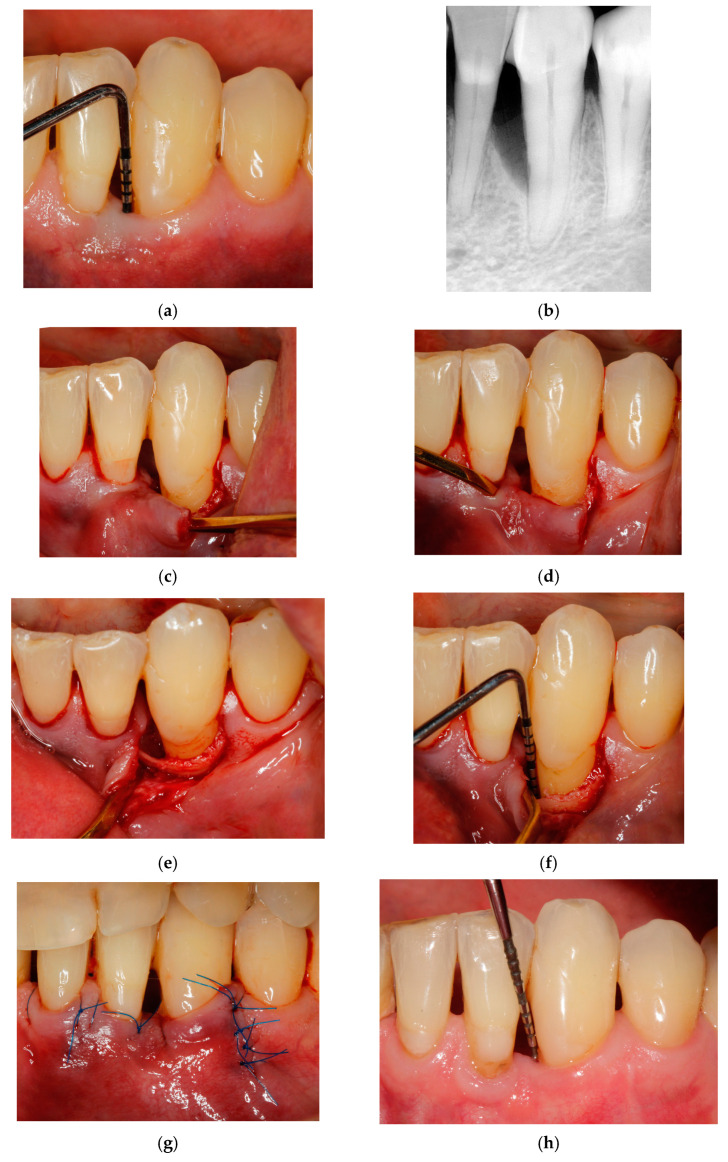

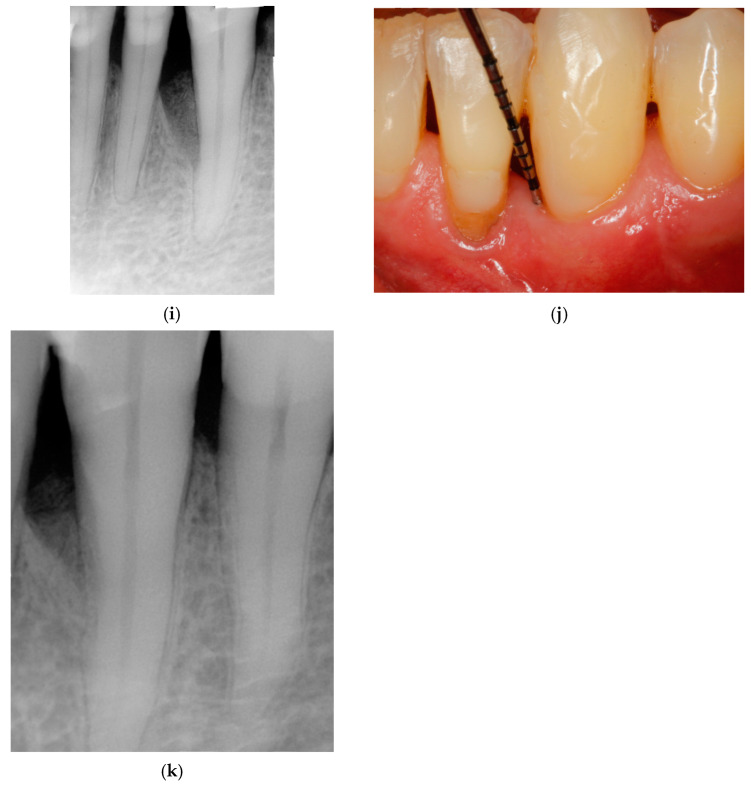
Representative case 1. (**a**) Baseline radiographic view. (**b**) Baseline clinical measurements. (**c**) A single vertical incision was made at the distal aspect of the mandibular left canine. (**d**) The flap was elevated, and the interdental tunnel was created by carefully dissecting beneath the papilla adjacent to the defect. (**e**,**f**) A clinical view of the intrabony defect after debridement. (**g**) the primary closure of the surgical area with interrupted sutures over the vertical incision and single sling suture. (**h**,**i**) Clinical and radiographic views at 6 months postsurgery. (**j**,**k**) Clinical and radiographic views 3 years after the surgery.

Schematic illustrations of the described technique are presented in Figure 1, whereas Figure 2 shows one representative case clinically and radiographically at the baseline, at the time of the surgery and subsequently after 6 months and 3 years.

## 3. Results

### 3.1. Statistical Analysis

All data were collected and analyzed at the Department of Periodontology, Medical University of Warsaw. Data analysis was conducted using a statistical software package (Statistica 13). Each patient contributed only one intrabony defect to the study. The variables PD, CAL, GR, KTA and DD were expressed in millimeters, and FMPS and FMBS were expressed in percentages, while the radiographic angles were reported in degrees. The mean and standard deviation (SD) was calculated for each parameter. The assumption of normal distribution was checked for all parameters by means of the Shapiro–Wilk test. The intra-group analyses for the variables FMPS, FMBS, PD, CAL, GR, KTW and DD were carried out using a paired *t*-test or Wilcoxon signed-rank test accordingly. Each comparison was tested separately and refers to specific time points (e.g., baseline vs. 6 months (P1), 6 months vs. 3 years (P2) and baseline vs. 3 years (P3)). A statistically significant difference was set at *p*-value < 0.05 and should be interpreted for specific intervals during this study.

### 3.2. Results

Fifteen patients (eight women and seven men) with an average age of 42.61 ± 6.94 years, ranging from 30 to 57 years, were included in this study, with each contributing one intrabony defect. The baseline defect characteristics are summarized in Table 1. At 6 months and at 3 years, all patients with one intrabony defect each were available for the analysis. All patients completed the study without any dropouts. During the follow-up, no adverse events were recorded, and no teeth were lost.

At the initial assessment, the average FMPS and FMBS values remained below 15%, reflecting a high standard of oral hygiene and minimal residual infection. Among the 15 identified intrabony defects, the majority were located in esthetically significant areas, including 3 in the incisors, 7 in the canines, 6 in the premolars and 2 in the molars. The defects were classified into three categories: four presented as one-wall defects, seven as two-wall and seven as three-wall. At baseline, the mean FMPS and mean FMBS values were below 15%, indicating good oral hygiene and low levels of residual infection. Healing after the surgery was uneventful in all patients. Complete gingival wound closure was accomplished for all defect sites. All participants completed the 6-month follow-up. The mean FMPS and FMBS values did not change significantly from baseline. The following changes in the assessed variables were observed: a PPD reduction of 4.33 ± 1.25 mm (*p* < 0.0001), CAL gain of 4.87 ± 1.36 mm (*p* < 0.0001) and DD reduction of 4.27 ± 1.19 mm (*p* < 0.0001) (Table 1). The changes in GR and KTW between baseline and after 6 months were not statistically significant. No formation of scar tissue was detected.

At the secondary assessment 3 years postsurgery, the results were the following. All patients exhibited changes in FMPS and FMBS over time. FMPS increased from approximately 11.93% at baseline to 14.13% at 6 months and further to 22.67% at 3 years. FMBS followed a similar trend, increasing continuously from 11.07% to 13.07% at 6 months and reaching 18.75 at 3 years. (Table 1). These differences over time were statistically significant (*p* < 0.05) for both P2 and P3. In terms of changes in PPD, a significant reduction in PPD was observed at 6 months and 3 years in both groups (*p* < 0.05). PPD decreased from 7.03 ± 1.61 mm at baseline to 2.70 ± 0.70 mm at 6 months and then increased to 3.33 ± 0.89 mm at 3 years. The differences between the time points were statistically significant (*p* < 0.0001 for both P1 and P3). CAL decreased from approx. 7.60 ± 1.92 mm at baseline to 2.73 ± 1.44 mm at 6 months and then to 3.08 ± 1.16 mm at 3 years. The changes between these time points were statistically significant (*p* < 0.001 for both P1 and P3). Significant changes in DD were observed for intervals P1 and P3 (*p* < 0.05). DD decreased from 4.59± 1.24 mm at baseline to 0.32 ± 0.35 mm at 6 months and then increased to 0.38 ± 0.31 mm at 3 years. The reductions were statistically significant between two of the points (*p* < 0.001 for both comparisons from baseline). Significant changes in BF% and LDF were shown over time (*p* < 0.05). BF% decreased from approximately 92.48% at 6 months to 92.00% at 3 years. LDF followed a similar trend, decreasing from approximately 4.325 mm at 6 months to 4.10 mm at 3 years. The minor decreases in both parameters were statistically significant for LDF (*p* = 0.0236) and not significant for BF (*p* = 0.1332). The changes in GR and KTW between 6 months and 3 years were not statistically significant.

## 4. Discussion

Maintaining papilla integrity and the soft tissue profile has tremendous value in periodontal regenerative surgery, especially in the esthetic area. In the presented results, the modified EPPT significantly reduced PPD, improved CAL and decreased DD. No statistically significant differences were observed in regard to GR and KTW. Modification included extending the buccal flap elevation mesiodistally to expose the cortical bone around the defect at the adjacent tooth to enable better access to the surgical area. Moreover, trauma to the interdental papilla was minimized, indicating that the described technique was minimally invasive. This technique is especially valuable in cases of a narrow interdental space, a thin periodontal phenotype, a reduced papilla, or extensive bone loss. The findings of this study are analyzed in comparison with the existing literature on the EPPT introduced by Aslan et al. [13,14,23] and our previous research, which included a 6-month follow-up assessment [24]. The prospective case series by Aslan et al. in 2021 [23] reported that the EPPT with a combination of EMD and deproteinized bovine bone mineral resulted in a statistically significant PPD reduction and CAL gain, as well as a negligible GR increase. A gain in clinical attachment level (CAL) may be achieved through the preservation of the integrity of the papilla, which enables space and stability for the blood clot. The healing phase was uneventful in all cases, and primary wound closure was obtained in all cases. The data from this research points towards the conclusion that the EPPT with a collagen barrier and bone substitutes facilitated uninterrupted wound healing. This finding provides strong evidence for the high potential of this distinct flap design to enhance wound stability and healing, even in the presence of a collagen membrane [23]. Authors in the literature suggest that the proper indication for the EPPT is a 2-wall intrabony defect with a missing buccal wall and a relatively well-preserved lingual wall [12,13,14,23]. In our study, the majority of the defects were 2-wall and 3-wall; however, by using our modification of the EPTT and extending the flap mesiodistally, access was enhanced even for two single-wall intrabony defects. Moreover, to optimize access and improve the visualization of a single-wall intrabony defect, the length of the vertical releasing incision can be extended. Furthermore, a significant radiographic improvement was achieved, as indicated by the defect fill and bone defect depth (DD). The bone grafts used in this study were radiation-sterilized with a dose of 35 kGy in an accelerator with the use of a high-speed electron beam [21]. Radiation-sterilized deep-frozen bone allografts demonstrate osteoinductive properties and are removed faster than lyophilized irradiated bone [25]. Various allogeneic bone substitutes have demonstrated the ability to promote the regeneration of intrabony defects [24,25,26]. For instance, Majzoub et al. [27] reported a mean clinical attachment level (CAL) gain of 3.55 ± 1.85 mm and a probing pocket depth (PPD) reduction of 3.87 ± 1.87 mm one year after guided tissue regeneration (GTR) using either freeze-dried or solvent-dehydrated bone allografts. The meta-analysis by Trombelli et al. suggests that the combination of enamel matrix derivative (EMD) and bone grafting may be advantageous in the treatment of unsupported deep bone defects [28]. However, a multi-center randomized controlled clinical trial by Tonetti et al. indicated that when combined with a minimally invasive flap, EMD showed a 269% higher success rate in achieving a ≥3 mm CAL gain in 3-wall defects with papilla preservation flaps compared to 1-wall defects [29]. Postoperative gingival recession is an adverse outcome of surgical intervention for intrabony defects and may be associated with the morphological characteristics of bone dehiscence. By preserving the volume of intact supracrestal soft tissues through the avoidance of papilla incision, as demonstrated in the presented technique, postsurgical flap shrinkage is effectively minimized. This is particularly crucial in esthetic regions, where minimizing postsurgical gingival recession is essential, as esthetic preservation represents a key objective of surgical periodontal therapy [30]. In a meta-analysis of randomized controlled trials by Graziani et al., it was mentioned that flap surgeries for intrabony defect treatment are associated with an average increase in recession depth of 1.15 mm at 1 year postsurgery [31]. Moreover, according to Vandana et al., regions with a thin gingival biotype or reduced keratinized tissue exhibit lower resistance to recession following surgical trauma [32]. Even though microsurgical techniques are recommended for their ability to enhance visualization of the surgical field and to allow atraumatic flap manipulation, according to Rasperini et al., gingival recession and increased recession depth following periodontal regeneration remain a relatively common occurrence [33]. In the present study, there was not only a lack of an increase in gingival recession after surgical therapy but there was also a slight improvement in gingival recession reduction and an increase in keratinized tissue width observed; however, these changes did not reach statistical significance. In recent years, many authors have contributed to the development of a technique for periodontal regeneration that aims to maintain intact or even coronally advance the gingival margin, such as the “soft tissue wall technique” proposed by Rasperini et al. [34], the “entire papilla preservation” technique by Aslan et al. [12], modified vestibular incision subperiosteal tunnel access (M- VISTA) [35] and the “non-incised papillae surgical approach” (NIPSA) [36]. In order to improve root coverage in intrabony defect treatment, techniques based on connective tissue grafts (CTGs) have been suggested [37]. Nevertheless, there is a considerable paucity of well-conducted randomized controlled trials (RCTs) in the literature to objectively compare and assess the reported techniques. No conclusive evidence supports the superiority of one method over another. Therefore, further comparative studies involving the EPPT and the presented MEPPT are essential to elucidate their true benefits and their relevance to established approaches. To consider treatment outcomes, Trombelli et al. [38] proposed a suitable endpoint for implementing the treat-to-target approach in studies assessing the effectiveness of active periodontal therapy. The authors defined the targeted endpoint as a postsurgery PD ≤ 4 mm in the short-term. A recent systemic review and meta-analysis by Aimetti et al. [39] reported that GTR compared to PPTs achieved a higher probability of pocket resolution. However, the use of non-resorbable membranes over the past years has been declining due to their high complication rates. Therefore, studies tend to corroborate that newer, minimally invasive techniques may enhance wound stability due to reduced flap extension and minimal interdental tissue elevation, thereby raising doubts about the added value of incorporating supportive biomaterials for regeneration [40]. Justification for the modified EPPT therapy may be challenging if comparable outcomes can be achieved using standard techniques. In the systematic review and meta-analysis by Pasqualini et al. [41], the authors investigated clinical periodontal parameters (PPD, CAL and gingival recession) after treatment using the Minimally Invasive Surgical Technique (MIST), Modified Minimally Invasive Surgical Technique (M-MIST) and/or any technique for papilla preservation, such as Entire Papilla Preservation (EPP), the modified-papilla preservation technique (M-PPT) or the simplified-papilla preservation technique (SPPT). Their conclusions indicated that MIST, M-MIST and papilla preservation techniques demonstrate significant efficacy in improving periodontal conditions in intrabony defect sites while maintaining minimal patient morbidity. However, modifications to the entire papilla preservation technique could offer advantages in the surgical management of certain isolated intrabony defects in terms of flap design, wound healing, better visualization, minimal morbidity and similar, satisfactory clinical results at the same time. One of the main surgical challenges is to achieve sufficient access and visibility of the intraosseous defect to perform precise instrumentation while minimizing trauma to the interproximal papilla. At the same time, the use of magnification and microsurgical instruments plays a crucial role in addressing these difficulties. Any minimally invasive regenerative procedure is a technique-sensitive approach, and any damage to the papilla resulting from a suboptimal surgical technique may negatively affect regenerative outcomes. The findings of this study indicate that the modified EPPT, combined with EMD and allogenic bone grafting, is an effective approach for treating intrabony defects, demonstrating statistically significant and sustained clinical improvements over a 3-year period. However, it is important to note that the outcomes are derived from a single treatment modality without a control group, limiting the ability to draw definitive conclusions about the proposed approach and its superiority over previously established techniques. Further follow-up clinical trials are necessary to determine the contribution of each component utilized in the presented procedure to the overall results and to validate the presented findings.

## 5. Conclusions

Within the limitation of this case series, it can be concluded that the proposed modification of the entire papilla preservation technique might be beneficial in the surgical treatment of isolated intrabony defects. Although the findings are encouraging, further research involving larger cohorts and more diverse patient populations is necessary to confirm the long-term clinical efficacy of the modified EPPT.

## 6. Study Limitations

A limitation of this study is the sample population recruited for the trial, as it was selected based on strict eligibility criteria. However, it is important to note that a significant proportion of patients with severe periodontitis are smokers and may also present with systemic comorbidities such as diabetes. Therefore, further research involving a more diverse patient population is required to establish the efficacy and reliability of the modified entire papilla preservation technique (M-EPPT) as a regenerative approach for the treatment of periodontitis.

## Figures and Tables

**Table 1:** Baseline parameters and 6-month post-treatment clinical outcomes.

Variables	Baseline (Mean ± SD)	95% CI	6 Months (Mean ± SD)	95% CI	3 Years (Mean ± SD)	95% CI	Baseline-6 Months (Mean ± SD)	95% CI	P1	6 Months–3 Years (Mean ± SD)	95% CI	P2	Baseline-3 Years (Mean ± SD)	95% CI	P3
Tooth type, *n*															
Incisor	3														
Canine	7														
Premolar	3														
Molar	2														
Tooth position, *n*															
Maxilla	7														
Mandible	8														
FMPS, %	11.93 ± 4.08	9.67, 14.19	14.13 ± 3.81	12.02, 16.25	22.67 ± 9.31	6.59, 15.80	+2.20 ± 3.36	−4.06, −0.34	0.0487	8.42 ± 9.12	−14.21, −2.62	0.0085	10.58 ± 7.70	−15.48, −5.69	0.0006
FMBS, %	11.07 ± 5.12	8.23, 13.90	13.07 ± 4.01	10.85, 15.29	18.75 ± 4.77	3.38, 8.10	+2.00 ± 3.16	−3.75, −0.25	0.1113	6.08 ± 4.21	−8.76, −3.41	0.0004	8.00 ± 4.77	−11.51, −4.49	0.0004
PPD, mm	7.03 ± 1.61	6.14, 7.92	2.70 ± 0.70	2.31, 3.09	3.33 ± 0.89	0.63, 1.51	−4.33 ± 1.25	3.64, 5.02	<0.0001	0.58 ± 0.79	−1.09, −0.08	0.0271	−3.63 ± 1.75	2.52, 7.73	<0.0001
CAL, mm	7.60 ± 1.92	6.54, 8.66	2.73 ± 1.44	1.94, 3.53	3.08 ± 1.16	0.82, 1.98	−4.87 ± 1.36	4.12, 5.62	<0.0001	0.25 ± 1.14	−0.97, 0.47	0.4627	−4.42 ± 1.73	3.32, 5.52	<0.0001
GR, mm	0.67 ± 0.82	0.21, 1.12	0.63 ± 0.85	0.16, 1.11	0.33 ± 0.65	0.46, 1.11	−0.03 ± 0.48	−0.23, 0.30	0.9905	0.17 ± 0.94	−0.76, 0.43	0.5505	0.08 ± 0.90	−0.66, 0.49	0.7545
KTW, mm	3.27 ± 1.16	2.62, 3.91	3.17 ± 1.19	2.51, 3.83	3.50 ± 1.09	0.77, 1.85	+0.10 ± 0.28	−0.06, 0.26	0.9339	−0.08 ± 0.29	-	-	−0.08 ± 0.29	−0.10, 0.27	0.3388
Intrabony depth, mm	5.37 ± 1.86	4.34, 6.39													
Intrabony width, mm	2.33 ± 0.84	1.87, 2.80													
Main defect configuration, *n*															
One-wall	4														
Two-wall	7														
Three-wall	7														
DD, mm	4.59 ± 1.24	3.90, 5.27	0.32 ± 0.35	0.12, 0.52	0.38 ± 0.31	0.22, 0.52	−4.27 ± 1.19	3.61, 4.93	<0.0001	0.09 ± 0.25	0.25, 0.07	0.2308	−4.18 ± 1.15	3.45, 4.92	<0.0001
Radiographic angle, degrees	32.99 ± 8.28	28.41, 37.58													
LDF, mm			4.325 ± 1.147	3.60, 5.04	4.10 ± 1.28	0.90, 2.17				−0.28 ± 0.37	0.05, 0.52	0.0236			
BF, %			92.48 ± 7.83	86.74, 98.22	92.00 ± 5.95	4.21, 10.10				−2.00 ± 4.27	−0.72, 4.72	0.1332			

CAL = clinical attachment level; DD = radiographic defect depth; FMBS = full-mouth bleeding score; FMPS = full-mouth plaque score; GR = gingival recession; KTW = keratinized tissue width; PPD = probing pocket depth. In the Difference column (mean ± SD), growth is indicated with a “+” symbol, and loss is indicated with a “−” symbol.

## Data Availability

The corresponding author will share the data upon request due to legal and ethical reasons.

## Abbreviations

FMPS%	Full-mouth plaque score
FMBS%	Full-mouth bleeding score
PPD, mm	Probing pocket depth (measured in millimeters)
CAL, mm	Clinical attachment level (measured in millimeters)
GR, mm	Gingival recession (measured in millimeters)
KTW, mm	Keratinized tissue width (measured in millimeters)
DD, mm	Radiographic defect depth (measured in millimeters)
LDF, mm	Linear defect fill
BF, %	Bone-fill percentage
